# Abstracts

**Published:** 1912-08

**Authors:** 


Boehm in *The Theraputic Gazette* (Jan 15, 1912.) In acute systemic gonorrhœa I firmly believe that all cases should receive the combined vaccine treatment, but in simple acute urethral gonorrhœa either the plain gonococcus or combined vaccine in many cases is harmful instead of beneficial, because the circulation is already overloaded with toxic products and vaccine treatment may act to the detriment of the patient.

Lydston; *Medical Review of Reviews,* (Jan. 1912) Offers the following objections to the reporting of venereal diseases.

1. The venereal diseases are easily concealed.

2. The impulse to conceal them on the part of the victims is practically impossible to control.

3. Opportunity would be offered to the political and official blackmailer, whose pickings are already fat enough.

4. Under the present social conditions the victim of venereal disease would be the butt of ridicule, practically ostracised socially and treated very much as a leper might be.

5. On account of this attitude of society toward the venereal diseases those who would suffer most severely from social suspicion, repugnance or ostracism would be those who have innocently contracted the disease.

6. Physicians in general would be likely to break the laws by concealing their patients ailments. It is my opinion that most high-minded physicians would go to jail rather than betray the confidences of their patients. The physician who would expose the ills of an innocent woman, or young girl especially, to public scorn and aversion, would be unworthy of his calling. Once the reporting began however, no descrimination could justly be made by the authorities; innocently and viciously infected alike would be subject to report.

7. Patients would often go to quacks, resort to patent medicines, or do without treatment altogether, rather than consult a reputable physician who might conform to the law and report the case.

